# Relationship Between Family Support, C-Reactive Protein and Body Mass Index Among Outpatients with Schizophrenia

**DOI:** 10.3390/healthcare13141754

**Published:** 2025-07-20

**Authors:** Argyro Pachi, Athanasios Tselebis, Evgenia Kavourgia, Nikolaos Soultanis, Dimitrios Kasimis, Christos Sikaras, Spyros Baras, Ioannis Ilias

**Affiliations:** 1 Psychiatric Department, “Sotiria” General Hospital of Chest Diseases, 11527 Athens, Greece; irapah67@gmail.com (A.P.); ekavourgia@gmail.com (E.K.); soultanisnikolaos@gmail.com (N.S.); kasimisdimitrios@yahoo.com (D.K.);; 2 Nursing Department, Sotiria Thoracic Diseases Hospital of Athens, 11527 Athens, Greece; cris.sikaras@gmail.com; 3 Department of Endocrinology, Hippokration Hospital, 11527 Athens, Greece; iiliasmd@yahoo.com

**Keywords:** family support, C-reactive protein, Body Mass Index, schizophrenia, obesity

## Abstract

**Background/Objectives:** Schizophrenia has been associated with increased inflammatory and metabolic disturbances. Perceived family support potentially affects inflammatory and metabolic biomarkers. The aim of this study was to determine the interrelations between family support, C-reactive protein (CRP) and Body Mass Index (BMI) in a sample of outpatients with schizophrenia. Importantly, this study sought to elucidate the effect of perceived family support on inflammatory processes among patients with schizophrenia. **Methods:** In this cross-sectional correlation study, 206 outpatients with schizophrenia in clinical remission completed a standardized self-report questionnaire that assessed family support (Family Support Scale—FSS). Sociodemographic, clinical and laboratory data were also recorded. **Results:** Among the participants, 49.5% had detectable CRP values (≥0.11 mg/dL), whereas 14.6% had positive CRP levels (>0.6 mg/dL). There was a significant difference in CRP levels among the different BMI groups (normal weight/overweight vs. obese). For obese patients, the crude odds ratios (ORs) for detectable and positive CRP values were 1.980 (95% confidence interval (CI) [1.056, 3.713]) and 27.818 (95% CI [6.300, 122.838]), respectively. Significant positive correlations were observed among CRP, BMI and illness duration, while scores on the FSS were negatively associated with these variables. The results of binary logistic regression analysis indicated that both BMI and family support were significant factors in determining the likelihood of having positive CRP levels, with each unit increase in the BMI associated with a 17% (95% CI [0.025, 0.337]) increase in the odds, and with each unit increase in family support leading to an 8.6% (95% CI [0.018, 0.15]) decrease. A moderation analysis revealed that the association between family support and the probability of having positive CRP levels depends on the BMI value, but only for obese patients did the protective effect of family support significantly decrease the magnitude of the risk of having positive CRP (b = −0.1972, SE = 0.053, OR = 0.821, *p* = 0.000, 95% CI [−0.3010, −0.0934]). **Conclusions:** The effect of perceived family support on inflammatory responses becomes evident in cases where beyond metabolic complications, inflammatory processes have already been established. Increased perceived family support seems to protect against inflammation and, notably, the association between low perceived family support and increased inflammation is even stronger. Establishing the role of family involvement during the treatment of patients with schizophrenia through inflammatory processes is a novelty of this study, emphasizing the need to incorporate family therapy into psychiatric treatment plans. However, primary interventions are considered necessary for patients with schizophrenia in order to maintain their BMI within normal limits and avoid the subsequent nosological sequelae.

## 1. Introduction

Contemporary scientific evidence implicates inflammatory processes in the pathogenesis of schizophrenia [1,2,3], with C-reactive protein (CRP) representing one of the most frequently investigated biomarkers for systemic inflammation [4,5]. CRP is a nonspecific acute-phase protein that is primarily generated by liver cells in both acute and chronic inflammation in response to inflammatory cytokines [6]. A number of cross-sectional studies have demonstrated increased CRP levels in schizophrenia patients compared to controls [7,8,9], and longitudinal studies have demonstrated that higher CRP levels at baseline may increase the risk of schizophrenia at follow-up [10,11]. However, while observational data show a positive association between CRP and schizophrenia risk, Mendelian Randomization (MR) analyses indicate that genetically elevated CRP protects against schizophrenia risk [12,13,14]. A hypothesis positing that a deficient immune response early in life could lead to chronic infection and the subsequent increased risk of schizophrenia has been proposed as a plausible explanation to reconcile this inconsistency [15,16], but no definitive conclusions that disentangle this discrepancy have been reached [17].

Research studies have shown that CRP levels in patients with schizophrenia are increased during acute phases [18,19], as well as in cases with more severe psychopathology [20,21], treatment-resistant cases [22] and antipsychotic-free disorder [23], and are associated with positive [24] and negative symptoms [25,26], cognitive deficits [27] and the risk of metabolic syndrome [28]. Metabolic disturbances and obesity are highly prevalent among patients with schizophrenia [29,30,31], with obesity affecting more than 50% of these patients [29]. Body Mass Index (BMI) is frequently used as an index of obesity, and its predictive utility for the occurrence of metabolic syndrome in patients with schizophrenia has been confirmed [32]. According to research in drug-naïve patients with schizophrenia, BMI and the rate of obesity increase in parallel with the duration and course of the disorder [33]. Many factors contribute to elevated BMI in patients with schizophrenia, including disorder-specific symptoms, lifestyle elements and socioeconomic issues, such as negative symptoms, social withdrawal, lack of physical activity, insufficient exercise, inadequate sleep, unhealthy diet and psychotropic medications [34,35,36]. The correlation between schizophrenia and BMI has been investigated in genetic and epidemiological studies [37,38,39,40], and recent research has revealed a significant causal association between genetically predicted childhood BMI and the subsequent risk of schizophrenia in adulthood through an MR framework [41].

The positive association between BMI and CRP levels in the general population has been established [42,43], and the higher CRP values observed in individuals with an increased BMI are linked to a state of low-grade systemic inflammation that affects overweight and obese individuals [44,45]. Research findings indicate that being overweight significantly raises the likelihood of clinically relevant increases in CRP, and this link is particularly pronounced in individuals with obesity [46,47]. MR studies suggest a directional relationship between BMI and CRP levels, whereas a bidirectional association has not been confirmed [43,48]. Among patients with schizophrenia, research studies have documented a positive association between BMI and CRP levels; however, the directional relationship between these biomarkers remains elusive due to the cross-sectional nature of most of these studies [28,49]. A recent longitudinal study indicated a bidirectional relationship between BMI and CRP, with the effect of CRP on future BMI being more potent than vice versa [50]. More specifically, research findings suggest that, assuming equal adiposity, patients with schizophrenia may have higher levels of inflammation compared to healthy controls [11]. Thus, inflammation and metabolic syndrome in schizophrenia have been a focus of scientific interest, and individuals with schizophrenia consistently show several metabolic disruptions and have increased systemic inflammation, with both having effects on the brain and contributing to higher morbidity and premature mortality associated with the disorder [51,52,53,54,55,56].

Research evidence suggests that the inflammatory response may serve as the final common pathway through which environmental risk factors are implicated in the pathophysiology of schizophrenia [57,58,59]. Social support through relationships with family and significant others, possibly through beneficial influences on immune-mediated inflammatory processes, has been identified as a protective factor associated with lower rates of morbidity and mortality [60,61]. Perceived social support, which is assumed to be a more representative aspect for appraising the quality of support received, protects against the risks of inflammation [62]. Perceived family support is considered an essential element of social support and refers to how an individual views the assistance provided by family members. Most people with schizophrenia live with family members and, even for those who live separately, their families are actively involved in their lives, providing care and support [63,64]. Parental support significantly influences the development of coping strategies for individuals with schizophrenia [65]. The existing literature reveals associations between parenting behaviors and child inflammatory markers, suggesting that positive parenting is associated with lower levels of inflammation, while negative parenting is linked to higher levels of inflammation [66]. In addition, a higher level of parental support was associated with decreased inflammation in children, especially when medical conditions were present [67]. Concerning the role of parent–child attachment in obesity, a meta-analytic review demonstrated that BMI was negatively related to secure attachment [68]. Moreover, insecure and disorganized attachment in early infancy has been associated with higher CRP levels in early childhood and predicted later obesity compared to children with secure attachment [69]. In addition, elevated CRP levels and BMI were documented in adults with schizophrenia and a history of childhood maltreatment [70].

Increased physical morbidity and premature mortality among patients with schizophrenia, mostly due to the higher prevalence of cardiometabolic disorders, have been extensively studied [71,72]. These disorders are associated with increased levels of circulating inflammatory markers, such as CPR [73]. Such low-grade inflammation and metabolic alterations have also been consistently observed among patients with schizophrenia [74,75,76], and both inflammatory and metabolic indices may serve as predictors of clinical outcome [52,77]. Recent research confirmed that the metabolic disturbances recorded in patients with schizophrenia are mainly attributed to schizophrenia-induced obesity [78]. A large-scale multicenter study provided evidence that individuals with both obesity and schizophrenia exhibited more pronounced neurostructural brain alterations than people with only one of these conditions [51]. Research findings have identified a shared genetic liability between binge eating behaviors and schizophrenia, with impaired social cognition as an intermediate phenotype [79]. In addition, disordered eating behaviors, such as emotional eating and loss-of-control eating, are prevalent among patients with schizophrenia and are linked with both obesity and inflammation [80,81,82,83]. In the search for a favorable factor that would counteract these adverse conditions, family support emerged, and several studies demonstrated that the active involvement and systematic engagement of family members in the care of patients with schizophrenia predicted positive outcomes by enhancing adaptation resources, improving treatment adherence and ultimately increasing the quality of life of the patients [84]. However, schizophrenia has a tendency to cause cognitive deficits in various domains, including social cognition [85,86], which could affect the ability to perceive family support [87] and possibly undermine its protective influence [88].

The theoretical framework of this study was that the perceived presence of supportive family relationships has the ability to protect individuals with schizophrenia from adverse inflammatory and metabolic pathophysiological processes, while the perceived absence of such relationships increases disease risks. A literature review did not identify any studies that explored the association between CRP and perceived family support and further examined whether this association differed along the BMI spectrum in a sample of outpatients with schizophrenia, leaving a knowledge gap that we sought to address. Therefore, the purpose of this study was to explore the interrelations between CRP, BMI and perceived family support among individuals with schizophrenia in outpatient treatment. Notably, this study sought to clarify the effect of perceived family support on CRP levels among patients with schizophrenia. More specifically, elucidating the extent to which the association between CRP and perceived family support is stratified by BMI could improve our understanding of the role of perceived family support and provide opportunities for psychotherapeutic and psychosocial interventions. The abovementioned assumptions give rise to the following hypotheses:

**Hypothesis 1.**  *BMI is positively associated with and predicts CRP.*

**Hypothesis 2.**  *Perceived family support is negatively related to and predicts CRP*.

**Hypothesis 3.**  *The interaction between BMI and perceived family support serves as a moderator of the association between CRP and perceived family support. The moderating effect of BMI is assumed to be most pronounced in obese individuals who have higher levels of low-grade inflammation due to their increased BMI*.

## 2. Subjects and Methods

### 2.1. Research Design

A cross-sectional correlational design study was conducted to address the above hypotheses. Participants were outpatients with a diagnosis of schizophrenia who received treatment at the Outpatient Psychiatric Department of “Sotiria” General Hospital from October 2022 to March 2024. After approval from the Clinical Research Ethics Committee of “Sotiria” General Hospital (Approval Number: 26741/21-10-2021), the researchers explained the research objectives to the participants, who provided both written and verbal informed consent. This study was performed in accordance with the World Medical Association Declaration of Helsinki (1975, revised 2008), following the ethical principles outlined in the General Data Protection Regulation (GDPR-2016/679) of the European Union, and the guidelines of the International Committee of Medical Journal Editors. Prior to the performance of any procedures related to this study and according to the ethical considerations, patients were assured that any information obtained would remain confidential, that participation in the survey was completely voluntary and that potential volunteers could opt to withdraw from the study at any point. Once each participant had received comprehensive information on the study procedure and enrolled, an appointment was arranged for the following morning between 8:00 and 9:00 a.m. to collect demographic and clinical data, as well as weight and height measurements, and then proceed to laboratory examinations. Before venous blood was drawn, patients were requested to fast for more than eight hours and to maintain a normal diet the night before, avoid alcohol consumption and refrain from strenuous exercise. At the end of the appointment, each participant was required to complete a semi-structured form created by research staff to gather demographic information and to respond to a self-report questionnaire regarding their perception of family support.

### 2.2. Study Participants

A purposive sampling method was used to perform the study, which included 206 outpatients with a confirmed psychiatric diagnosis of schizophrenia, based on the International Classification of Diseases-10 (ICD-10), who were receiving ongoing treatment at the Psychiatric Outpatients Department. Participants had to fulfill the following inclusion and exclusion criteria: (i) being between 18 and 65 years old, (ii) being in a stable psychiatric condition, being in clinical remission, and not having been hospitalized or had changes in psychotropic medication or psychosocial status within 90 days before joining the study, (iii) having been hospitalized for psychiatric issues at least twice before (for diagnostic accuracy), (iv) having a coherent verbal rapport during the completion of the data questionnaire. Participants were excluded if they had untreated visual or hearing impairments, neurological disorders or damage to the central nervous system, developmental disabilities, signs of intellectual disability, severe cognitive and neuropsychological impairment, personality disorders, psychotic disorders associated with clinical medical conditions or substance use, substance addiction and history of substance use in the last six months, and a record of current substance or alcohol abuse. Included participants were preliminarily assessed by a physician to determine the presence of any clinically significant or unstable medical disorders or chronic general medical conditions. Patients with past or present cardiovascular disease, chronic lung disease, liver, kidney disease, arthritis or rheumatoid arthritis, autoimmune, blood diseases, diabetes mellitus and/or HbA1C levels above 5.7%, pregnancy or lactation were excluded. In addition, patients were excluded from the study in the following cases: active infectious illness or primary inflammatory disease; current use of corticosteroids or non-steroidal anti-inflammatory drugs; recent or ongoing use of warfarin or anticoagulant medications; recent or current use of antidepressants; use of antibiotics or probiotics in the past three months; a urine drug screen positive for psychoactive drugs; smoking habits (more than 1 cigarette per day); and drinking habits (more than 1 unit alcohol per week). To confirm the above criteria, upon study enrollment, a thorough health assessment and clinical evaluation were performed.

### 2.3. Minimum Sample Size Calculation

The sample size calculation for the statistical analysis of the binary logistic regression was carried out using the G-Power 3.1 software [89]. To compute the required sample size for the most influential independent variable, which is BMI, the significance level was set to 0.05, the statistical power to 0.95, and the R^2^ for the other x variables to 0.218 (which is the value calculated by regressing the independent variable of prime interest on all other independent variables using multiple linear regression) [90], selecting the normal distribution type for BMI, with 28.8493 as the mean value and 4.86474 as the standard deviation, 1.293 as the odds ratio and the probability of y = 1 under H0 as 0.0000689541, which is estimated by using the formula EXP(B)/(1 + EXP(B)), with B being the constant intercept estimate B (see Section 3). The total sample size determined using this calculation was 115, with an actual power of 0.95; thus, the sample size in this study was deemed adequate. The G-Power software was also used to verify sample adequacy for moderation analysis. To compute the required sample size for regression with the R square increase, the significance level was set to 0.05 and the statistical power to 0.95, with the number of tested predictors at 1, the total number of predictors at 3 and the effect size determined with the formula f^2^ = R_2_^2^ − R_1_^2^/1 − R_2_^2^ = 0.08. The total sample size determined using this calculation was 152, with an actual power of 0.95; thus, the sample size in this study was also deemed adequate.

### 2.4. Measurement Tools

The participants in this study provided demographic data and clinical information such as age, gender and illness duration.

#### 2.4.1. BMI

BMI measurements were conducted by trained nurse personnel. Based on a standard formula, each participant’s weight (in kilograms) was divided by their height (in squared meters) to determine their BMI. These measurements were performed with the SECA 769 electronic scale with height measuring rod (SECA, Hamburg, DE, Germany). According to the World Health Organization criteria, the sample was grouped into underweight with BMI < 18.5 kg/m^2^, normal weight with 18.5 ≤ BMI < 25 kg/m^2^, overweight with 25 ≤ BMI < 30 kg/m^2^ and obesity with BMI ≥ 30 kg/m^2^.

#### 2.4.2. CRP

Serum CRP levels were determined using a quantitative turbidimetric test (CRP-turbilatex) with the latex agglutination immuno-turbidimetric method. The lowest detectable limit was 0.11 mg/dL, and the highest normal reference value was 0.6 mg/dL, according to the manufacturer’s instructions (SPINREACT 2021, Spinreact, S.A.U. Girona, Spain) and the laboratory reference range. The maximum reference level for CRP was set to 1 mg/dL because values above this threshold tend to indicate a suspected infection [91]. The analysis was performed at the clinical biochemistry laboratory of “Sotiria” General Hospital.

#### 2.4.3. Family Support Scale (FSS)

The Greek version of the Family Support Scale is designed to measure the sense of support individuals receive from family members with whom they reside. The scale is self-administered and includes 13 items on a Likert scale, with 1 denoting ‘strongly disagree’ and 5 indicating ‘strongly agree’. All items revolve around the relationships among cohabiting individuals [92,93,94]. A greater sense of family support is indicated by higher ratings on the scale. People living independently were not required to complete the scale [92]. Cronbach’s alpha for internal reliability was 0.786 for this study.

### 2.5. Statistical Analysis

Initially, a descriptive analysis was carried out. Means and standard deviations are used to express continuous variables, and percentages are used to report categorical variables. A Kolmogorov–Smirnov test was used to assess the distribution of data. Skewed variables (illness duration, BMI and scores on FSS) were transformed to normal with the two-step approach [95]. The distribution of CRP was markedly right-skewed and was thus treated as a non-normally distributed variable, which was assessed with nonparametric methods. Using the Kruskal–Wallis test, differences in CRP levels among BMI groups were evaluated. The independent-samples *t*-test was employed to compare illness duration, scores on the FSS and BMI differences according to gender. To compare CRP values according to gender, we used the Mann–Whitney U-test. Correlations between continuous variables were determined using Spearman’s correlation test for nonparametric bivariate correlation analyses (including CRP) or using Pearson correlations for normally distributed continuous variables (excluding CRP). Further, CRP was categorized as a dichotomous variable: detectable but normal CRP values (≥0.11 mg/dL) and positive CRP values (>0.6 mg/dL). Unadjusted odds ratios (ORs) and 95% confidence intervals (95% CIs) for detectable and positive CRP values for participants with obesity relative to those without obesity were estimated using univariable binary logistic regression. To assess the risk factors for positive CRP values in patients with schizophrenia, a binary logistic regression analysis was performed, adjusted for the effects of possible confounding variables (age, gender, illness duration, BMI and scores on FSS). Moderation by BMI was assessed through the introduction of a BMI*FSS interaction term in the logistic regression analysis. Simple slope analysis was performed using Hayes’ SPSS Process Macro Model 1 to report the moderating effect at different BMI levels. The data analyses were conducted using the SPSS software (Version 24.0). For all statistical analyses, statistical significance was set to *p* < 0.05 (two-tailed).

## 3. Results

### 3.1. General Characteristics of Participants and Scores on Outcome Variables

**Figure 1:**
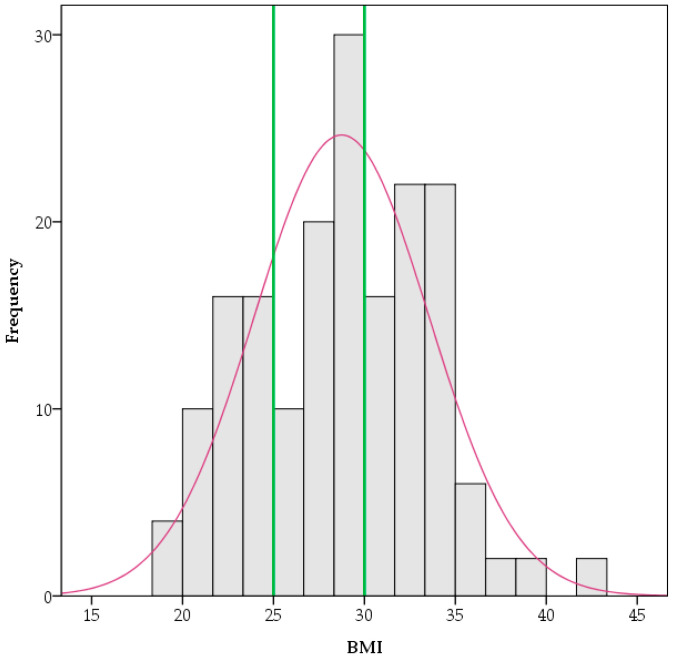
The distribution histogram of BMI among participants. Vertical green lines represent cutoffs for WHO BMI categories at 25 and 30 kg/m^2^.

This study included a total of 206 participants (100 males and 106 females). According to the BMI values, 46 participants (22.3%) were classified as normal weight, 60 (29.1%) as overweight and 72 (35%) as obese. The distribution histogram of BMI among participants is presented in Figure 1. There were no underweight participants in the study, and the sample’s mean weight was within the overweight category. Among the participants, 49.5% had detectable CRP values, whereas 14.6% had positive CRP levels. Their demographic and clinical characteristics are presented in Table 1. Regarding gender, no differences were observed in the study variables except for age, with females being older compared to males (*t*-test *p* < 0.05, 44.98 ± 14.37 vs. 41.36 ± 10.79, Table 1).

**Figure 2:**
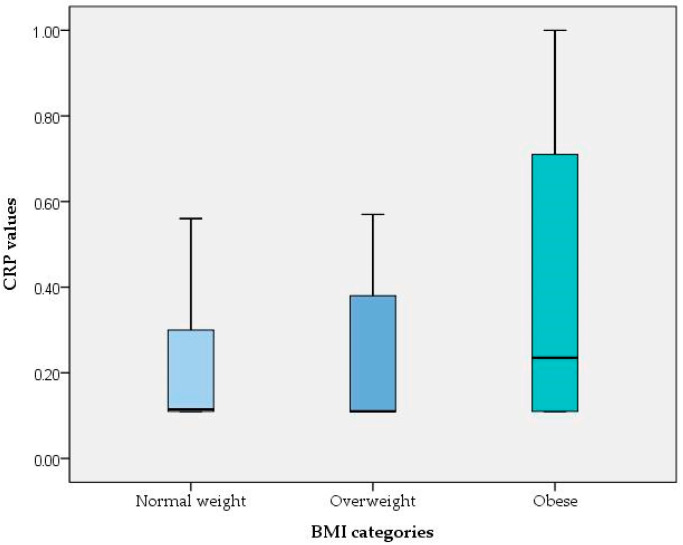
CRP values for the different BMI categories.

The median and the IQR of CRP values for normal-weight participants were 0.1150 and 0.19, respectively; for overweight participants, the corresponding values were 0.11 and 0.27, while for obese participants, these values were 0.2350 and 0.60. These results indicate that there are differences in the medians across the different BMI groups (normal-weight/overweight vs. obese) as well as in the spread of the data, suggesting that a median test would be insufficient to describe the important features of the data. A Kruskal–Wallis test indicated significant differences in CRP values among the different BMI groups (χ^2^(2) = 12.07, *p* = 0.002), with a mean rank CRP score of 75.27 for normal weight, 75.36 for overweight and 100.76 for obese (Figure 2).

### 3.2. Correlations Among Continuous Variables

Pearson’s correlation showed significant associations among normally distributed variables. Illness duration was positively correlated with BMI value and negatively correlated with the FSS score. In addition, age was positively correlated with illness duration and the BMI value (Table 2). Since CRP values were non-normally distributed, Spearman’s correlation was employed to determine these relationships. Specifically, CRP had a positive correlation with BMI and a negative association with the FSS score (Table 3).

### 3.3. Binary Logistic Regression Analyses

To determine whether obese participants were significantly more likely to have detectable and positive CRP values compared to non-obese participants, we performed simple binary (univariable) logistic regression analysis and calculated the unadjusted odds ratios (ORs) and 95% confidence intervals (95% CIs) for detectable and positive CRP values for obese compared to non-obese participants. The results indicate that obese patients are 1.980 times more likely (OR) to have detectable CRP values (95% confidence interval (CI) [1.056, 3.713]) and 27.818 times more likely (OR) to have positive CRP values (95% CI [6.300, 122.838]) compared to non-obese participants (Table 4 and Table 5).

A binary logistic regression analysis was conducted to investigate the effects of associated factors (age, gender, illness duration, BMI, FSS score) on the likelihood of having positive CRP values (>0.6 mg/dL). Ensuring the reliability and validity of this method necessitates checking that the underlying assumptions are satisfied: the independence of observations (no autocorrelation or repeated measures, and the calculated Durbin–Watson value of 2.140 was within the normal range); linearity of the logit, which was confirmed with the Box–Tidwell procedure (the relationship between the continuous predictor variables and the log odds of the binary outcome was linear); the absence of multicollinearity among the independent variables (checked through the correlation method: the correlation coefficients were <0.70 and the VIF values were within the acceptable range); and the absence of significant outliers based on Cook’s distance (<4/206).

The model was statistically significant (x^2^(5) = 29.379, *p* < 0.001), accounting for 22% (Cox and Snell R-Square) and 38.4% (Nagelkerke R-Square) of the variance in the presence of positive CRP values and correctly classifying 89.8% of cases. The Hosmer and Lemeshow test suggested a good fit to the data (x^2^(8) = 13.555, *p* = 0.094). In the model, BMI was a significant predictor (B = 0.158, Wald = 5.396, *p* = 0.020, Exp(B) = 1.171, 95% CL [1.025, 1.337]), with each unit increase associated with a 17% increase in the odds of having positive CRP values. At the same time, family support was also a significant predictor (B = −0.090, Wald = 6.086, *p* = 0.014, Exp(B) = 0.914, 95% CL [0.850, 0.982]), with each unit increase in family support leading to a 8.6% (95% CI [0.018, 0.15]) decrease in the odds of having positive CRP values (Table 6). These results indicate that both BMI and family support are significant risk and protective factors, respectively, which determine the likelihood of the presence of positive CRP values, thus confirming the first and second research hypotheses.

### 3.4. Moderated Binary Logistic Regression Analysis

**Figure 3:**
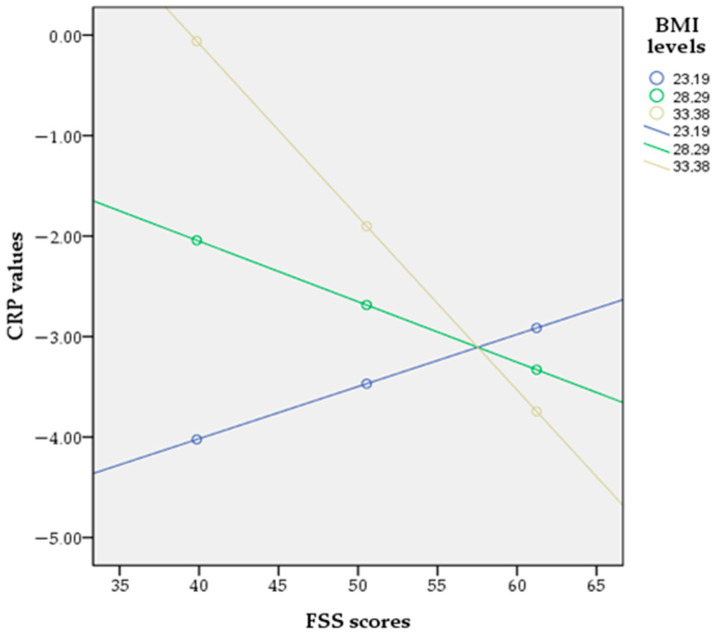
Simple slopes indicating the interaction in the moderation analysis.

**Figure 4:**
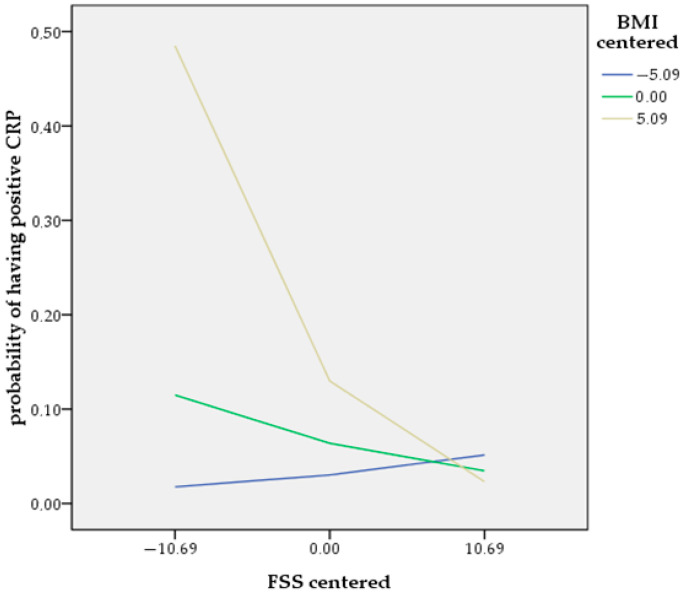
A line graph of the interaction between family support and BMI as predictors of the probability of having positive CRP.

Further, a moderated binary logistic regression analysis was conducted utilizing Model 1 of Hayes’ PROCESS macro to investigate whether the relationship between family support and the likelihood of having positive CRP values was moderated by BMI. The dependent variable was the probability of having positive CRP, the independent variable was family support, the moderator was BMI, and the included covariates were age, gender and illness duration. The interaction term was significant (B = −0.022, SE = 0.0100, *p* < 0.05), indicating that the effect of family support on the probability of having positive CRP values depended on the BMI level (Table 7), thus confirming the third hypothesis. Simple slopes analysis revealed that at high BMI levels (+1 SD), family support was negatively associated with the likelihood of having positive CRP (B = −0.1724, *p* < 0.01), whereas at low BMI levels (−1 SD), the association was not significant (B = 0.0519, *p* = 0.46) (Table 8, Figure 3). The model explained 45% of the variance in positive CRP values (Nagelkerke R^2^ = 0.4521). In Figure 4, to visualize the nature of the BMI-by-family-support interaction as predictors of the probability of having positive CRP and help with the interpretation, we used the centered scores of both predictors.

## 4. Discussion

This study investigates the association between perceived family support, circulating CRP and BMI, and the results of the correlation analysis, binary logistic regression and moderation analysis provide strong evidence confirming the research hypotheses. More specifically, concerning the first hypothesis, the positive correlation between circulating CRP and measured BMI and the role of BMI as a risk factor increasing the likelihood of having positive CRP values is consistent with findings from previous studies conducted with participants from the general population and among patients with schizophrenia [42,43,44,45,46,47,48,49]. Most of these studies suggest that the observed correlation between CRP and BMI is attributed to increased BMI, with CRP being an index of elevated adiposity [45,47,48,96,97,98]. In particular, the pathophysiological process in obesity and metabolic dysregulation implicates systemic inflammation through the activation of macrophages in the adipose tissue, which release inflammatory cytokines, causing hyperlipidemia and insulin resistance. This further worsens the metabolic status, which, in turn, promotes the production of cytokines by the adipose tissue, thus contributing to chronic inflammation [99,100,101].

The investigation of the second and third research hypotheses led to the main findings of this study, which highlight the protective role of perceived family support among outpatients with schizophrenia, being able to counteract the low-grade inflammation by reducing the likelihood of positive CRP values. Most individuals with schizophrenia, due to the disabilities of the disorder, rely on family members as their primary caregivers. Clinical evidence suggests that the vast majority of patients with schizophrenia are unable to maintain basic social functions, and only a minority retain regular employment [102,103]. Thus, the existence of a supportive family network is of utmost importance. Supportive family relationships, beyond ensuring treatment adherence, may motivate healthy behaviors that promote good health and improve overall well-being. Healthy diet and physical exercise are well-known anti-inflammatory factors [104,105] and are frequently encouraged by a protective family environment, whereas pro-inflammatory diets [106], smoking and/or drug abuse are usually deterred.

Evidence from a large general adult population study documented that social/family strain significantly increased the risk of inflammation, while social/family support attenuated inflammation [107]. In particular, the aforementioned study demonstrated that the harmful effects of social/family strain on inflammatory processes were more potent than the beneficial effects of social/family support on inflammation [107]. These results are consistent with our findings and provide a plausible explanation for the steeper slope in the relationship between the odds of having positive CRP values and expressing lower-than-average perceived family support, while a less steep slope characterizes the relationship between the odds of having positive CRP values and scoring above average on the FSS (Figure 4). Another finding from this study that needs to be clarified is that the protective anti-inflammatory effect of family support is significant only for obese patients. In agreement with other studies with participants from the general population [108,109] and among patients with schizophrenia [28,52,110], obese participants in this study were significantly more likely to have positive CRP values compared to non-obese individuals. In these cases, where beyond metabolic complications, inflammatory processes have already been established, family support has the ability to attenuate this low-grade inflammation. Yet, because this study is cross-sectional, precluding the confirmation of the direction of causality, these findings could signify that low perceived family support, as experienced by patients with strained and adverse family backgrounds, may have resulted in increased levels of inflammation.

Studies indicate that greater social strain is linked to higher levels of inflammation [111,112]. Meanwhile, a meta-analysis suggested that social support and social integration are significantly associated with lower levels of inflammation [113,114]. Research indicates that individuals with schizophrenia have lower perceived social support than those without schizophrenia [88], and frequently, early-life adversities force them to withdraw and isolate themselves from other family members [115]. However, due to the highly prevalent social cognitive deficits among these patients, their families are the main, if not the only, social network [116]. Their role is to compensate for key social stressors and to protect the emotional health of their vulnerable relatives by combating stigma, enhancing self-esteem and coping strategies, reducing feelings of isolation, increasing social engagement, and promoting a sense of belonging. On the other hand, family strain derived from high levels of expressed emotion, conflictual family relationships, stigma, and emotional and financial burdens significantly impacts the mental health of individuals with schizophrenia who experience higher levels of distress, leading to increases in pro-inflammatory responses that ultimately lead to poorer treatment outcomes [117,118].

As stated, strain from family relationships may play an important role in inflammatory processes, because relationships with family members are likely to last through significant periods of life and are not a matter of choice, as in other relationships. When family relationships are particularly stressful, the need to maintain them over time may increase the strain caused by these relationships, making family tension detrimental to health, which manifests in inflammatory processes. In turn, immune dysregulation may lead to a cluster of chronic metabolic disorders [119]. For instance, research has shown that inflammation can interfere with insulin production and may also cause hypothalamic dysfunction, both of which are involved in weight gain [120,121]. Furthermore, changes in lipid and lipoprotein metabolism linked to inflammation lead to raised triglyceride levels and lowered HDL cholesterol synthesis [122]. From another perspective, the literature suggests that disordered eating behaviors often co-exist with psychotic disorders, and social cognitive deficits are particularly prevalent among these patients [79], rendering them less able to appreciate family support. In addition, research studies have already documented cognitive deficits and related neuropsychiatric symptoms associated with obesity, as well as emotional eating behaviors in an attempt to regulate negative emotions [81,123].

More than one-third of participants in this study were obese. Findings from other studies among outpatients with schizophrenia report comparable or even higher prevalence rates of obesity [29,124,125]. The relationship between schizophrenia and obesity has gained attention due to the high risk of associated comorbidities, such as cardiovascular diseases and metabolic syndrome, increasing all-cause morbidity and mortality and reducing life expectancy [126,127]. Several studies have reported associations between obesity and negative symptoms, insomnia and night-eating and a decrease in quality of life among individuals with schizophrenia [128,129,130,131]. An understanding of the clinical factors that predict obesity risk is critical for targeting patients with psychotic disorders who are more prone to weight gain and metabolic syndrome [132]. Patients with schizophrenia are frequently characterized by a lack of control overeating behaviors and are more likely to consume unhealthy foods [133]. Several risk factors are implicated in these problematic eating behaviors and include tobacco smoking, type 2 diabetes, sleep disturbances, adverse effects of psychotropics, anxiety and depression [134,135,136,137], and lack of psychosocial rehabilitation [80,138].

Overall, this study confirmed the three research hypotheses stated in the Introduction, and the results were discussed in conjunction with other relevant literature. Meanwhile, to highlight the novel findings, we also provide the explanatory pathway we followed to reach these conclusions. Therefore, in this study, we aimed to clarify the effect of perceived family support on inflammatory processes, as measured by CRP levels, among patients with schizophrenia. First, we confirmed that increased BMI was associated with elevated CRP values; specifically, each unit increase in BMI was associated with a 17% increase in the odds of positive CRP levels. On the other hand, perceived family support was negatively associated with CRP levels; in particular, each unit increase in perceived family support resulted in an 8.6% reduction in the odds of positive CRP levels. In order to determine the differential effect of high and low perceived family support on the odds of positive CRP values among patients with schizophrenia, we focused on obese participants, because in this study, obese patients were significantly more likely to have positive CRP values compared to non-obese participants. In this state of low-grade inflammation, expressing higher-than-average perceived family support decreases the probability of positive CRP levels. Moreover, the association between the odds of having positive CRP values and expressing lower-than-average perceived family support is even stronger. However, since this study is cross-sectional, the above results could indicate that low perceived family support may have resulted in an increased probability of positive CRP levels, which, in turn, could have been involved in weight gain. Future longitudinal studies may disentangle the direction of causality.

The abovementioned findings hold practical clinical implications. The fact that obesity and associated metabolic abnormalities are present even in the early stages of psychotic disorders suggests the need for early interventions. Standard protocols include effective metabolic monitoring guidelines and preventive measures for patients with schizophrenia, especially for patients on psychiatric medications, upon discharge from psychiatric hospitalization and during follow-up [139,140]. Lifestyle interventions, dietary manipulation and aerobic physical intervention programs lead to improvements in addressing obesity and preventing weight gain [141,142,143,144]. The most important finding from this study supports family involvement during the treatment of patients with schizophrenia. The National Institute for Health and Care Excellence (NICE) [145] incorporated family therapy into its clinical guidelines when drafting psychiatric treatment plans in order to provide psychoeducation, enable families to manage emotions effectively by reducing family strain, and improve their communication and problem-solving skills [146,147].

This study exhibits certain limitations. Due to the cross-sectional design, conclusions about the direction of causality cannot be drawn. Moreover, the generalizability of the overall results is limited because it was a single-center study in a specific geographic area. Another drawback was the lack of a control sample to compare the laboratory findings, but the aim of this study was to conduct a correlation analysis, not a comparative analysis. Additionally, data on nutrition and activity measures were not available. Similarly, although we had data on the prescribed antipsychotics and knowledge of their differential effects on the immune system, the sample size prevented subgroup analysis by antipsychotic type. In addition, there were no underweight participants in the study to identify possible differences in the results compared to the other participants. Finally, the results of this study might have been influenced by potential confounding factors, such as incidental psychosocial events or unknown medications administered to participants during the research period.

## 5. Conclusions

The results of this study elucidate the relationships between perceived family support, peripheral CRP and BMI and highlight the protective role of family support in moderating inflammatory processes, particularly in outpatients with schizophrenia who have a metabolic risk associated with obesity. Specifically, each unit increase in perceived family support resulted in an 8.6% reduction in the odds of positive CRP levels. Higher-than-average perceived family support reduces the likelihood of positive CRP levels among obese participants, and the association between the odds of having positive CRP values and the expression of lower-than-average perceived family support is even stronger. This study demonstrated the role of family engagement during the treatment of patients with schizophrenia through inflammatory processes. These findings support the integration of family therapy into psychiatric treatment plans, in addition to standard monitoring of weight and metabolic indices, to offer more promising results for prevention and intervention purposes.

## Figures and Tables

**Table 1:** Descriptive statistics of participants.

Gender		Age	Illness Duration (in Years)	BMI (kg/m^2^)	Family Support Scale (FSS)		CRP (mg/dL)
Male	Mean	41.36 *	14.44	29.24	51.03	Median	0.17
	N	100	80	90	82	N	94
	SD	10.79	11.01	4.22	10.62	IQR	0.30
Female	Mean	44.98 *	14.90	28.44	48.83	Median	0.19
	N	106	80	88	88	N	88
	S.D.	14.37	10.01	5.44	10.23	IQR	0.41
Total	Mean	43.22	13.98	28.85	49.89	Median	0.17
	N	206	160	178	170	N	182
	SD	12.85	11.97	4.86	10.45	IQR	0.39

* *t*-test *p* < 0.05; IQR: interquartile range. Notes: For illness duration, BMI and FSS, the means from the two-step transformation are presented.

**Table 2:** Correlations among age, illness duration, BMI and FSS.

Pearson Correlation N: 206		Age	Illness Duration (in Years)	BMI (kg/m^2^)
Illness duration (in years)	r	0.659 **		
	p	0.000		
	N	160		
BMI (kg/m^2^)	r	0.240 **	0.315 **	
	p	0.001	0.000	
	N	178	155	
Family Support Scale (FSS)	r	−0.151	−0.250	−0.125
	p	0.050	0.004	0.132
	N	170	152	156

** Pearson correlation *p* < 0.01.

**Table 3:** Correlations among CRP, age, illness duration, BMI and FSS.

Spearman’s Rho N: 206		Age	Illness Duration (in Years)	BMI (kg/m^2^)	Family Support Scale (FSS)
CRP	rho	0.140	0.038	0.213 **	−0.510 **
	p	0.060	0.638	0.005	0.000
	N	182	158	176	163

** Spearman’s rho correlations *p* < 0.01.

**Table 4:** Unadjusted OR values from univariable binary logistic regression analyses.

Dependent Variable: CRP Values ≥ 0.11 mg/dL (N: 176)						
Predictor	*B*	*SE*	*Wald*	*p*	OR	95% CL
Constant	−0.077	0.196	0.154	0.695	0.926	NA
BMI < 30 kg/m^2^	Ref.				Ref.	Ref.
BMI ≥ 30 kg/m^2^	0.683	0.321	4.534	0.033 *	1.980	(1.056, 3.713)

Notes: *B* = logistic regression coefficient; Ref. = reference category. Statistically significant at the * *p* < 0.05 level.

**Table 5:** Unadjusted OR values from univariable binary logistic regression analyses.

Dependent Variable: CRP Values > 0.6 mg/dL (N: 176)						
Predictor	*B*	*SE*	*Wald*	*p*	OR	95% CL
Constant	−3.932	0.714	30.324	0.000	0.020	NA
BMI < 30 kg/m^2^	Ref.				Ref.	Ref.
BMI ≥ 30 kg/m^2^	3.326	0.758	19.262	0.000 **	27.818	(6.300, 122.838)

Notes: *B* = logistic regression coefficient; Ref. = reference category. Statistically significant at the ** *p* < 0.01 level.

**Table 6:** Binary logistic regression analysis.

Dependent Variable: CRP Values > 0.6 mg/dL (N: 152)						
Predictor	*B*	*SE*	*Wald*	*p*	OR	95% CL
Constant	−2.024	2.741	0.545	0.000	0.460	NA
Age	−0.047	0.049	0.923	0.337	0.954	(0.866, 1.051)
Gender (Ref. = male)	0.552	0.616	0.805	0.370	1.738	(0.520, 5.811)
Illness duration	0.088	0.052	2.817	0.093	1.092	(0.985, 1.210)
BMI	0.158	0.068	5.396	0.020 *	1.171	(1.025, 1.337)
FSS	−0.090	0.037	6.086	0.014 *	0.914	(0.850, 0.982)

Notes: *B* = logistic regression coefficient; Ref. = reference category. Statistically significant at the * *p* < 0.05 level.

**Table 7:** Moderated binary logistic regression analysis.

Dependent Variable: CRP Values > 0.6 mg/dL (N: 152)								
Predictor	*B*	*SE*	*Wald*	*z*	*p*	Exp(B)	LLCL	ULCI
Constant	−34.5617	15.2349	5.1465	−2.2686	0.0233		−64.4215	−4.7020
FSS	0.5624	0.2951	3.6332	1.9061	0.0566	1.7548	−0.0159	1.1408
Moderator (BMI)	1.2664	0.5257	5.8028	2.4089	0.0160 *	3.5480	0.2360	2.2967
Interaction (FSS * BMI)	−0.0220	0.0100	4.8079	−2.1927	0.0283 *	0.9782	−0.0417	−0.0023
Covariates								
Age	−0.0687	0.0567	1.4648	−1.2103	0.2261	0.9336	−0.1799	0.0425
Gender	0.4568	0.6779	0.4541	0.6739	0.5004	1.5790	−0.8719	1.7856
Illness duration	0.0897	0.0597	2.2570	1.5023	0.1330	1.0938	−0.0273	0.2067

Notes: *B* = logistic regression coefficient. Statistically significant at the * *p* < 0.05 level.

**Table 8:** Conditional effects of FSS at different BMI levels.

BMI Level	Effect of FSS on Having Positive CRP Values	*SE*	*z*	*Wald*	*p*	LLCL	ULCI
Low (−1SD): 23.1927	0.0519	0.0709	0.7323	0.5362	0.4640	−0.0870	0.1908
Mean: 28.2865	−0.0602	0.0390	−1.5428	2.3802	0.1229	−0.1368	0.0163
High (+1SD): 33.3803	−0.1724	0.0571	−3.0197	9.1185	0.0025 *	−0.2842	−0.0605

Notes: Statistically significant at the * *p* < 0.05 level.

## Data Availability

The data and the questionnaires of the study are available upon request from the corresponding author.
